# Comparison of surface roughness and hardness of three different brands of esthetic coated NiTi archwires: invitro study

**DOI:** 10.1186/s12903-023-03497-8

**Published:** 2023-10-29

**Authors:** Amira A. Aboalnaga, Amal M. El Shahawi

**Affiliations:** 1 Department of Orthodontics and Dentofacial Orthopedics, Faculty of Dentistry, Cairo University, Cairo, Egypt; 2 Restorative and Dental Materials Department, Oral and Dental Research Institute, National Research Centre, Cairo, Egypt

**Keywords:** Coated archwires, Esthetic archwires, Epoxy resin coated archwires, Surface roughness, Surface hardness

## Abstract

**Background:**

The purpose of the current in vitro study was to evaluate the surface roughness and hardness of three brands of as-received esthetic coated NiTi archwires and compare them with the same parameters after immersion in artificial saliva.

**Methods:**

Three groups of 0.016 × 0.022 inch epoxy-coated NiTi orthodontic wires [Tooth tone coated NiTi (Ortho Technology, West Columbia, USA), EverWhite NiTi (American Orthodontics, Wisconsin, USA) and Nitanium Super Elastic Tooth Tone Plastic coated (Ortho Organizers, San Marcos, CA, USA)] were compared. Each group was subdivided into five as-received archwire specimens and five archwire specimens retrieved following immersion in artificial saliva for 28 days. Atomic force microscopy was used for analysis of average surface roughness (Sa). Hardness testing was performed using Digital Vickers hardness tester. ANOVA and Kruskal-Wallis tests were used for comparing the wire groups.

**Results:**

The ranking of (Sa) values was as follows: Nitanium Ortho Organizers > Everwhite American Orthodontics > Tooth tone Ortho Technology (P > 0.05). Nitanium Ortho Organizers archwires showed significantly greater (Sa) than both other groups following immersion in saliva (P < 0.001). The coating hardness of as-received and post-immersion archwires from Tooth tone Ortho Technology was significantly lower than the other groups (P < 0.001). For all the three types of archwires, the mean hardness of immersed wires was significantly lower than that of the as-received archwires (P < 0.001).

**Conclusions:**

Esthetic coated archwires have shown unpleasant surface changes following exposure to artificial saliva. These surface changes are affected by physical characteristics such as surface roughness and hardness of the coating.

## Background

Esthetic appearance of orthodontic appliances is one of the major concerns of our patients [1]. Most patients are willing to correct their malocclusion, however a large portion is not prepared to treat it with visible appliances [2]. In attempt to satisfy patients’ requirements, tooth-colored brackets and orthodontic wires have been introduced to the market [3, 4]. Tooth-colored orthodontic archwires are available in several forms; transparent non-metallic orthodontic wire Optiflex (Ormco Co., Glendora, CA), fiber-reinforced polymer archwire, and tooth-colored coated archwires [5]. Owing to the poor mechanical properties, the first two types of the esthetic archwires are not commonly used in clinical practice [6].

The tooth-colored coated archwires are enveloped with either epoxy resin or Teflon material [7]. The coating is applied in a depository process that covers the base wire, a process termed electrostatic coating [8]. Apart from the coating material, coated archwires differ in their coating thickness, scratch tendency and mechanical efficacy [9].

The surface roughness and hardness of the archwire’s coating are important parameters that affect the clinical performance of the archwire. Both parameters directly affect the surface topography of the archwire, which can critically modify its clinical efficiency. Increased surface roughness of an archwire can emphasize the friction coefficient and hence decrease the effectiveness of archwire-guided tooth movement [10]. Lower friction has a positive effect on the sliding movement between the wire and bracket [8], leading to accelerated tooth movement and superior anchorage control. Hardness of the coating material can influence its scratch and delamination tendency, which in turn affects surface contact area, corrosion behavior and biocompatibility of the archwires [11]. Besides, the latter parameters can impact the degree of plaque accumulation around the orthodontic appliances and liability of enamel to demineralization [12]. It should be pointed out that distortion and scratching of the coating material results in compromised esthetics and patient satisfaction [8].

In addition, it is essential that the coated archwire retains its surface characteristics when exposed to the oral environment. Electrolytic corrosion of orthodontic appliances due to the wet oral environment is inevitable [13]. The pH value of saliva, which ranges from 5.6 to 7.6, has a significant effect on the corrosion rate, which successively determine the archwire surface properties [14]. For clinical work, it is important to be aware of the possible consequences following exposure to various intraoral conditions. Therefore, the purpose of this study was to evaluate the surface roughness and hardness of as-received esthetic coated NiTi archwires and compare them with the same parameters after 28 days of immersion in artificial saliva.

## Methods

**Fig. 1:**
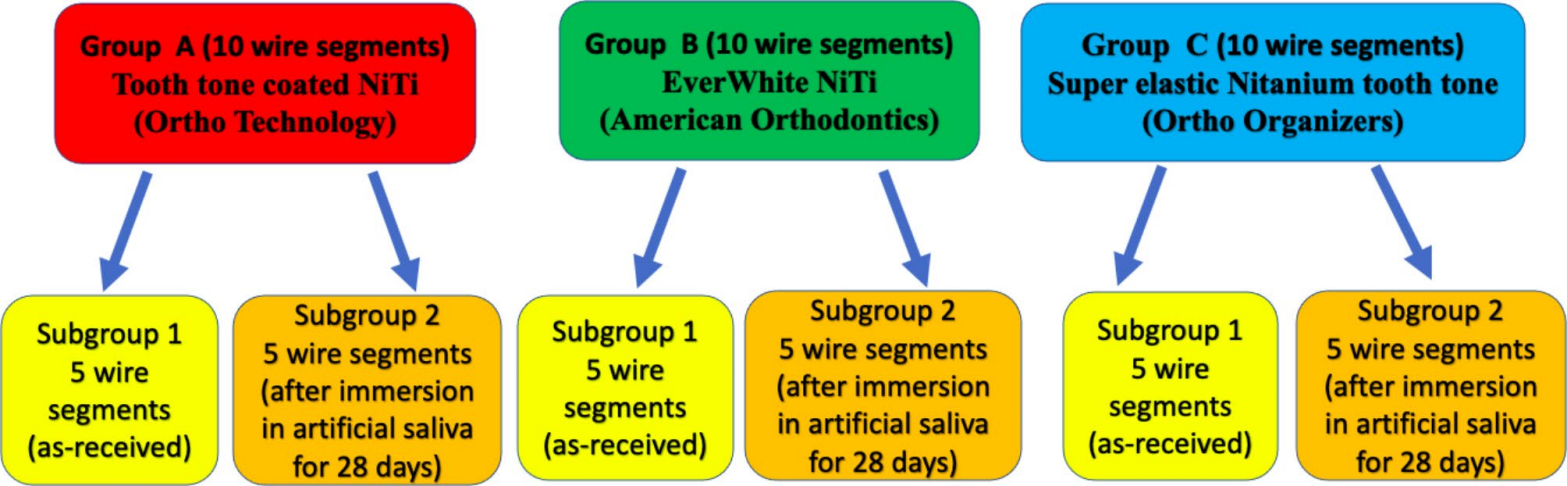
Grouping of archwire specimens for each measured variable

Three brands of esthetic white-coated 0.016 × 0.022 inch NiTi archwires were investigated in this study. Group A; Tooth tone coated NiTi (Ortho Technology, West Columbia, USA), Group B; EverWhite NiTi (American Orthodontics, Wisconsin, USA) and Group C; Super elastic Nitanium tooth tone (Ortho Organizers, San Marcos, CA, USA), all of which were fully coated archwires with an epoxy resin coating thickness of about 0.002 inch [8, 15–17]. Each group was subdivided into 2 subgroups as follows; subgroup 1: As-received archwire specimens and subgroup 2: Archwire specimens retrieved after immersion in artificial saliva (Fig. 1).

Based on a previous study [8], a sample size of five as-received and five immersed wire specimens per group was sufficient to detect a large effect size (f) = 0.88, with an actual power (1-β error) of 0.8 (80%) and a significance level (α error) 0.05 (5%) for two-sided hypothesis test.

### Specimen preparation

Five archwires in each group were cut into 4 segments using archwire cutter as follows; 2 anterior segments 5 mm in length each to be used for surface roughness measurement and two distal segments 15 mm in length to be used for hardness measurements. For each archwire group, 5 segments were measured as-received and 5 segments were immersed in artificial saliva for 28 days and then retrieved for measuring one variable.

Five archwire specimens of each group were immersed in individual test tubes containing 10 ml of artificial saliva fluid (2.38 g Na_2_HPO_4_, 0.19 g KH_2_PO_4_ and 8.00 g NaCl per liter of distilled water adjusted with phosphoric acid to pH 6.75). Each test tube was incubated in a water bath at 37 ^o^C for 28 days [14]. At the end of the incubation period, all archwire specimens were collected and washed with distilled water before being tested.

### Surface roughness test

Atomic force microscopy (AFM) was utilized for surface roughness analysis [4, 8]. A total number of 30 archwire specimens (n = 10/group) were prepared and divided as mentioned in the grouping of specimens (Fig. 1). Specimens 5 mm in length were evaluated using the AFM (Anton Paar GmbH-Tosca200 AFM, Germany) employing the tapping mode 500 micrometer increment with a rate of 1 line per second [4]. Average surface roughness (Sa) in nanometers was obtained by software analysis (Mountains 8.2 Software-Digital Surf, Besancon, France). The average surface roughness of each specimen was measured at three different sites and the mean (Sa) was then calculated.

### Hardness test

Hardness testing was performed using Digital Vickers hardness tester (NEXUS 4000 ^TM^, INNOVATEST, model no.4503, Netherlands) with force of 200 gms and a dwell time of 15 s [18]. A total number of 30 archwire specimens (n = 10/group) 15 mm in length were measured (Fig. 1). Three indentations were randomly made in each specimen’s surface. Each wire specimen was embedded in an acrylic resin mold to be properly fixed as the indenter is loaded vertically on it. The length of the impression diagonals was measured and the hardness was calculated as follows: Vickers hardness number (VHN) = 1.854P/d^2^, where P is the indentation load and d is the impression diagonal length. Three readings were taken from the center of each specimen, and the mean value was used as representative of the specimen.

### Statistical analyses

A standard software package (SPSS version 17.0, Chicago, Ill) was used for data analysis. Shapiro-Wilk test was used to test the normality hypothesis of variables to determine the appropriate parametric and non-parametric tests. Surface roughness (Sa) and Hardness (VHN) were described by the mean, standard deviation (SD), range (Minimum – Maximum), standard error (SE) and 95% confidence interval of the mean values. For normally distributed variables, ANOVA test was used for comparing the wire groups. In case of non-normally distributed variable, Kruskal-Wallis test was applied. Independent sample t test was used for comparing the as-received and immersed wires in each group. In all the above statistical tools, a probability value of 0.05 was considered significant.

## Results

### Surface roughness (Sa)

**Fig. 2:**
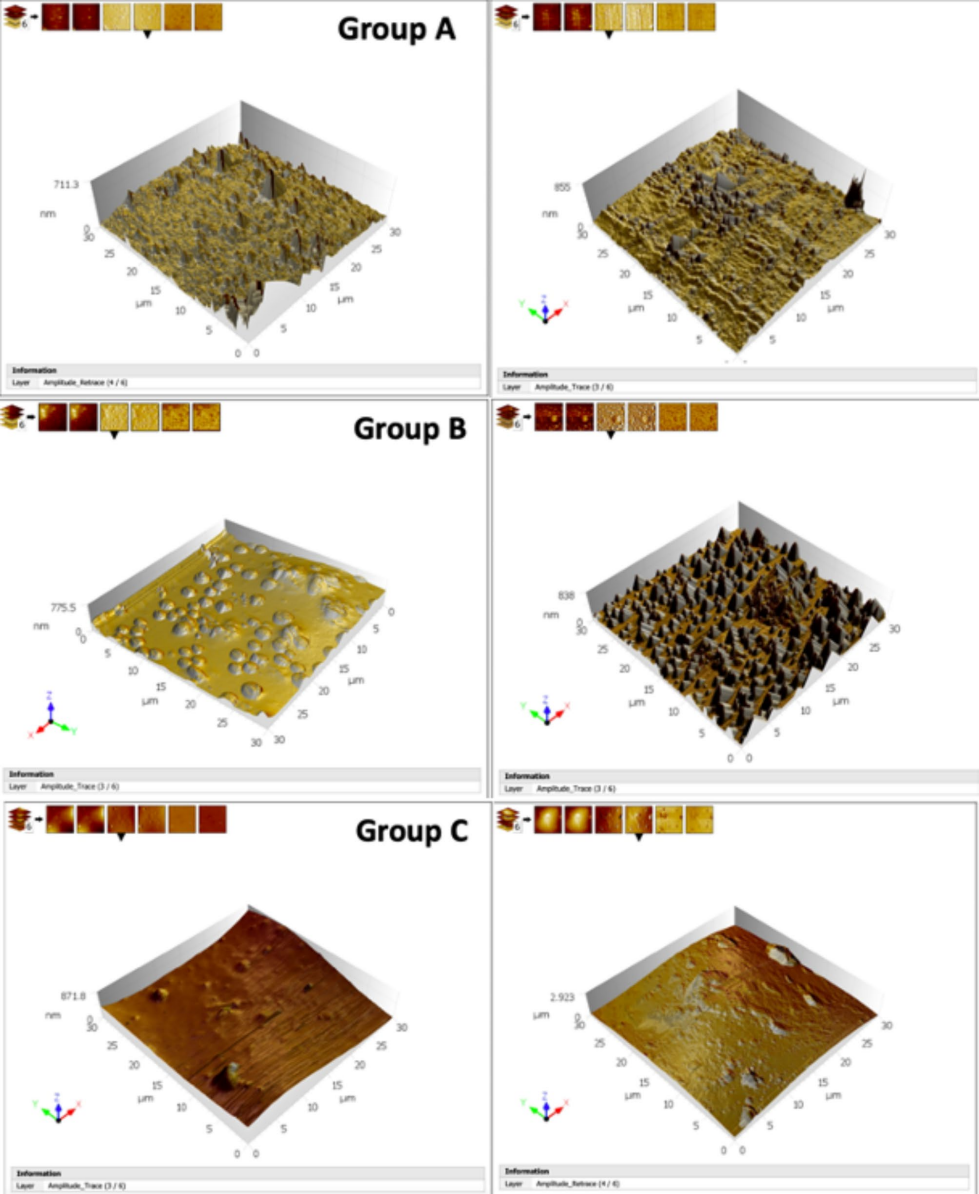
Tapping-mode AFM topographical 3D images of the as-received wire specimens (left photos) and post-immersion wire specimens (right photos) for the three wire groups respectively; (Group A: Tooth tone Ortho Technology, Group B: Everwhite American Orthodontics & Group C: Nitanium Ortho Organizers). Note that the post-immersion wire specimen of group C is demonstrated in micrometer (µm) due to the extreme surface irregularities

The tapping-mode AFM topographical images of the as-received and post-immersion wire specimens for the three groups is shown in (Fig. 2). Average surface roughness (Sa) of the as-received wire specimens is shown in (Table 1). Surface roughness results of as-received wires showed a non-parametric distribution, therefore Kruskal-Wallis test was used to compare between the groups. The ranking of (Sa) values from the highest to the lowest was as follows: Nitanium Ortho Organizers (group C) > Everwhite American Orthodontics (group B) > Tooth tone Ortho Technology (group A), however the difference was statistically non-significant (Table 1). The results of (Sa) after immersion in saliva showed a parametric distribution, therefore ANOVA test was used to compare between the groups. Nitanium Ortho Organizers (group C) showed significantly greater (Sa) than both other groups following immersion in saliva (Table 1). The comparison of surface roughness (Sa) between as-received and immersed wires in each group is shown in (Table 2). No significant difference was detected in groups A & B. The immersed archwire (Sa) was significantly higher than the as-received one in Nitanium Ortho Organizers archwires (group C).

**Table 1:** Average surface roughness (Sa) Mean, Standard deviation (SD), Standard Error (SE), 95% confidence interval of the mean and range (Minimum and Maximum) values of the as-received (Kruskal-Wallis test) and immersed wires (ANOVA test) in each group

Group		Mean (nm)	SD	SE	95% confidence interval of the mean		Minimum	Maximum	P value
					Lower bound	Upper bound			
As-received wires	Wire A	144.92	48	21.46	85.32	204.51	64.09	192.9	0.14178
	Wire B	153.06	70.51	31.53	65.52	240.61	60.22	224.3	
	Wire C	264.62	147.6	66.01	81.36	447.88	107.60	430.9	
Immersed wires	Wire A	130.13^a^	59.07	26.42	56.79	203.47	67.95	212.50	0.00001***
	Wire B	155.26^a^	37.33	16.70	108.91	201.61	109.50	202.40	
	Wire C	864.06^b^	248.85	111.29	555.07	1173.05	514.20	1217.00	

Wire A (Tooth tone Ortho Technology), Wire B (Everwhite American Orthodontics), Wire C (Nitanium Ortho Organizers), nm (Nanometer), ^a^ Different superscript letters indicate a statistically significant difference (P < 0.05), *** P < 0.001

**Table 2:** Independent sample t test for comparison of average surface roughness (Sa) of as-received and immersed wires in each group

Group		Mean (nm)	SD	SEM	Mean difference	SED	95% confidence interval of the mean		t	df	P value
							Lower bound	Upper bound			
Wire A	As-received wire	130.13	48.00	21.46	14.79	34.04	-63.70	93.28	0.43	8	0.67543
	Immersed wire	144.92	59.07	26.42							
Wire B	As-received wire	153.06	70.51	31.53	-2.20	35.68	-84.47	80.08	-0.06	8	0.95243
	Immersed wire	155.26	37.33	16.70							
Wire C	As-received wire	264.62	147.60	66.01	-599.44	129.39	-897.82	-301.06	-4.63	8	0.00168**
	Immersed wire	864.06	248.85	111.29							

Wire A (Tooth tone Ortho Technology), Wire B (Everwhite American Orthodontics), Wire C (Nitanium Ortho Organizers), nm (Nano meter), ** P < 0.01

### Hardness (VHN)

The results of hardness of as-received and immersed wires showed a parametric distribution, therefore ANOVA test was used to compare between the wire groups. The hardness of as-received wires from Tooth tone Ortho technology (group A) was significantly lower than the other groups (Table 3). Following immersion in saliva, archwires from Tooth tone Ortho technology (group A) showed significantly less hardness than the other groups (Table 3). For all the three types of wires, the mean hardness of immersed wires was significantly lower than that of the as-received wires (Table 4).

**Table 3:** Hardness (VHN) Mean, Standard deviation (SD), Standard Error (SE), 95% confidence interval of the mean and range (Minimum and Maximum) values of the as-received and immersed wires in each group (ANOVA test)

Group		Mean (VHN)	SD	SEM	95% confidence interval of the mean		Minimum	Maximum	P value
					Lower bound	Upper bound			
As-received wires	Wire A	22.17^a^	0.30	0.13	21.80	22.54	21.71	22.45	0.00001***
	Wire B	27.00^b^	1.23	0.55	25.47	28.53	25.07	28.17	
	Wire C	27.69^b^	1.05	0.47	26.38	28.99	26.90	29.11	
Immersed wires	Wire A	20.60^a^	0.43	0.19	20.07	21.13	19.85	20.88	0.00006***
	Wire B	21.73^b^	0.20	0.09	21.48	21.97	21.42	21.93	
	Wire C	22.21^b^	0.44	0.20	21.66	22.75	21.49	22.63	

Wire A (Tooth tone Ortho Technology), Wire B (Everwhite American Orthodontics), Wire C (Nitanium Ortho Organizers), nm (Nano meter). Different superscript letters indicate a statistically significant difference (P < 0.05). *** P < 0.001

**Table 4:** Independent samples t test for comparing mean hardness (VHN) of as-received and immersed wires in each group

Group		Mean (VHN)	SD	SEM	Mean difference	SED	95% confidence interval of the mean		t	df	P value
							Lower bound	Upper bound			
Wire A	As-received wire	22.17	0.30	0.13	1.57	0.23	1.03	2.11	6.76	8	0.00014***
	Immersed wire	20.60	0.43	0.19							
Wire B	As-received wire	27.00	1.23	0.55	5.27	0.56	3.98	6.56	9.44	8	0.0001***
	Immersed wire	21.73	0.20	0.09							
Wire C	As-received wire	27.69	1.05	0.47	5.48	0.51	4.31	6.65	10.78	8	0.0000***
	Immersed wire	22.21	0.44	0.20							

Wire A (Tooth tone Ortho Technology), Wire B (Everwhite American Orthodontics), Wire C (Nitanium Ortho Organizers), nm (Nano meter). *** P < 0.001.

## Discussion

Surface topography of an orthodontic archwire is known to influence its mechanical characteristics, esthetic appearance, corrosion behavior, and biocompatibility [7, 8]. Among the available techniques for evaluating surface roughness is the Atomic force microscopy (AFM), which is a non-invasive method for tentative analysis of surface roughness. It provides a three-dimensional insight into the wires’ micromorphology by presenting meticulous quantitative and qualitative assessment [10]. Despite the advantages of AFM analysis, there is the major drawback of a small scan size that impedes entire analysis of the sample [4].

Upon studying the qualitative surface topography of the as-received coated archwires, characteristic irregularities could be noted in the three groups. The pattern of coating surface roughness was unique for each wire group. Tooth tone Ortho technology archwires (group A) showed a spiky pattern of surface roughness, Everwhite American Orthodontics archwires (group B) showed a nodular pattern, whereas Nitanium Ortho Organizer archwires (group C) showed a striated type of surface roughness in addition to wide areas of concavities and convexities. The existence of particular surface features within the as-received polymer coating for each manufacturer has been reported previously [8, 19, 20]. Mousavi et al. [2] examined the as-received surface roughness of Everwhite American Orthodontics archwires and Nitanium Ortho Organizer archwires using AFM, and both brands revealed similar surface micro irregularities.

As regards the quantitative evaluation of surface roughness, Nitanium Ortho Organizers archwires revealed the highest average surface roughness (Sa) compared with the other two groups, yet the difference between as-received archwires was not significant. Likewise, Mousavi et al. [2] reported an insignificant difference in the surface roughness of four different coated archwires. Following immersion in artificial saliva, Nitanium Ortho Organizers was the only group which showed significantly greater coating surface roughness. However upon clinical use, surface roughness was shown to significantly increase in several tested coated archwires including Everwhite archwires [21]. The epoxy resin coating was reported to be unstable following exposure to saliva in several previous studies [15, 22–24] ranging from increased surface roughness to tearing and coating loss in multiple locations. This instability, which is manifested clinically as unpleasant discolouration and rupture of the coating layer [25], can be attributed to the hydrophilic property of resins [26]. When the epoxy resin is immersed in a solution, it absorbs water leading to bulging and cracking of its surface [27]. Faster deterioration was reported with increased acidity of saliva in patients with poor oral hygiene [15, 24]. Although the three wire brands had an epoxy resin coating, their behavior upon immersion in saliva differed. The present study emphasizes that manufacturer-specific variations in the coating composition influence the physical properties of coated archwires. Specific information about the processing of archwires is a property of each manufacturer and is not available.

Hardness value gives an indication about the coating’s resistance to scratching and plastic deformation [18]. The higher is the hardness of the coating layer, the greater is the resistance to plastic deformation, and hence better retention of the coating integrity and esthetics. Everwhite American Orthodontics and Nitanium Ortho Organizers as-received archwires had significantly greater hardness than Tooth toned Ortho Technology archwires. Nevertheless, all wires showed reduced hardness following exposure to saliva. Nitanium Ortho Organizers was formerly delineated to be most resistant to scratching compared to other coated archwires [11]. Esthetic coating of greater hardness will acquire less induced surface roughness upon clinical use. Although Nitanium Ortho Organizers archwires manifested the greatest average surface roughness, it also displayed the highest coating hardness, which may enhance its clinical performance.

Up to the present time, the influence of coating surface topography on the production of archwire-bracket friction remains controversial. The 3D model of surface roughness differed greatly among the brands and we cannot predict its influence on the resulting friction. The results of the present study are based on laboratory testing and therefore cannot precisely represent the clinical conditions.

Previous studies assured a positive correlation between the surface topography and friction [7]. Nevertheless the resulting friction in the intra-oral environment is a multifactorial subject affected by several physical and biological factors. Apart from the arch wire & bracket slot dimensions, form, and materials type [28], biological factors such as saliva, masticatory functions and oral hygiene can greatly impact the resulting friction, all of which are factors that cannot be controlled in experimental conditions [29]. Consequently, no clear correlation could be detected between surface roughness of the archwires and frictional resistance in other studies [30, 31].

It can be concluded that most coated archwires will eventually display undesirable surface changes following exposure to the intraoral environment. These surface changes are affected by physical characteristics such as surface roughness and hardness of the coating. Further studies are required to evaluate the effect of surface roughness and hardness of the coated archwire on the resulting friction. Moreover, in vivo studies addressing the effect of oral environment on the same parameters are required.

## Conclusions

The average surface roughness of the as-received archwires did not differ significantly among the three tested brands. Nitanium Ortho Organizers archwires showed significantly greater average surface roughness than Everwhite American Orthodontics and Tooth tone Ortho Technology archwires following exposure to artificial saliva. Everwhite American Orthodontics and Nitanium Ortho Organizers as-received wires showed significantly greater surface hardness than Tooth tone Ortho technology archwires. All three types of coated wire brands displayed significantly lower hardness values following exposure to artificial saliva. Within the limitations of this study, Everwhite American Orthodontics manifested better laboratory coating properties than the other two tested brands.

## Data Availability

The datasets used and analyzed during the current study are available from the corresponding author on reasonable request.

## Abbreviations

AFM: Atomic force microscopy
Sa: Average surface roughness
VHN: Vickers hardness number
